# Isolation and characterization of duck adenovirus 3 circulating in China

**DOI:** 10.1007/s00705-018-4105-2

**Published:** 2018-12-18

**Authors:** Shaohua Shi, Rongchang Liu, Chunhe Wan, Longfei Cheng, Zhen Chen, Guanghua Fu, Hongmei Chen, Qiuling Fu, Yu Huang

**Affiliations:** 1 Institute of Animal Husbandry and Veterinary, Fujian Academy of Agricultural Sciences, 247 Wusi Road, Fuzhou, Fujian People’s Republic of China; 2 Fujian Provincial Key Laboratory for Avian Diseases Control and Prevention, Fuzhou, Fujian People’s Republic of China; 3 Fujian Animal Diseases Control Technology Development Center, Fuzhou, Fujian People’s Republic of China

## Abstract

Recently, infectious disease outbreaks characterized by swelling and hemorrhagic liver and kidneys occurred in Muscovy ducklings in China. Four viruses were isolated and identified as adenoviruses by transmission electron microscopy (TEM) and polymerase chain reaction (PCR). Sequence analysis identified the new isolates as duck adenovirus 3 (DAdV-3), species *Duck aviadenovirus B*. The pathogenicity of the new isolate DAdV-3 FJGT01 was investigated using challenge experiments. The gross lesions in the animal experiment were similar to the clinical lesions observed in the diseased ducks. TEM examination of liver sample showed that virions accumulated and arranged in crystal lattice formations in the nuclei of hepatocytes. The present study provides new information about the epidemiology and characteristics of duck adenovirus associated with Muscovy ducklings.

**Electronic supplementary material:**

The online version of this article (10.1007/s00705-018-4105-2) contains supplementary material, which is available to authorized users.

## Introduction

According to the International Committee on Taxonomy of Viruses (ICTV), the family *Adenoviridae* includes five genera: *Atadenovirus, Aviadenovirus, Ichtadenovirus, Mastadenovirus* and *Siadenovirus* [1]. The genus *Aviadenovirus* includes 14 species: *Fowl aviadenovirus A-E* and *Goose aviadenovirus A*, *Duck aviadenovirus B*, *Pigeon aviadenovirus A-B, Falcon aviadenovirus A*, *Psittacine aviadenovirus B*, and *Turkey aviadenovirus B-D* [2]. So far, two duck adenoviruses (DAdVs) have been classified by the ICTV: DAdV-1 in the genus *Atadenovirus* and DAdV-2 in the genus *Aviadenovirus*. Waterfowl are known to be natural hosts of DAdV-1, and infections are usually asymptomatic [3]. In chickens and quail, DAdV-1 has been described as the causative agent of egg production problems [4, 5]. To our knowledge, infection with DAdV-2 has only been reported in ducklings. In 2014, Marek *et al.* published the complete genomic sequence of DAdV-2 GR isolated from Muscovy ducklings in France. Based on its genetic characteristics, DAdV-2 has been suggested to belong to the genus *Aviadenovirus* and the species *Duck aviadenovirus B* [6]. Another duck adenovirus was isolated almost simultaneously from Muscovy ducklings in China and then designated as a DAdV-3 candidate [7].

**Fig. 1:**
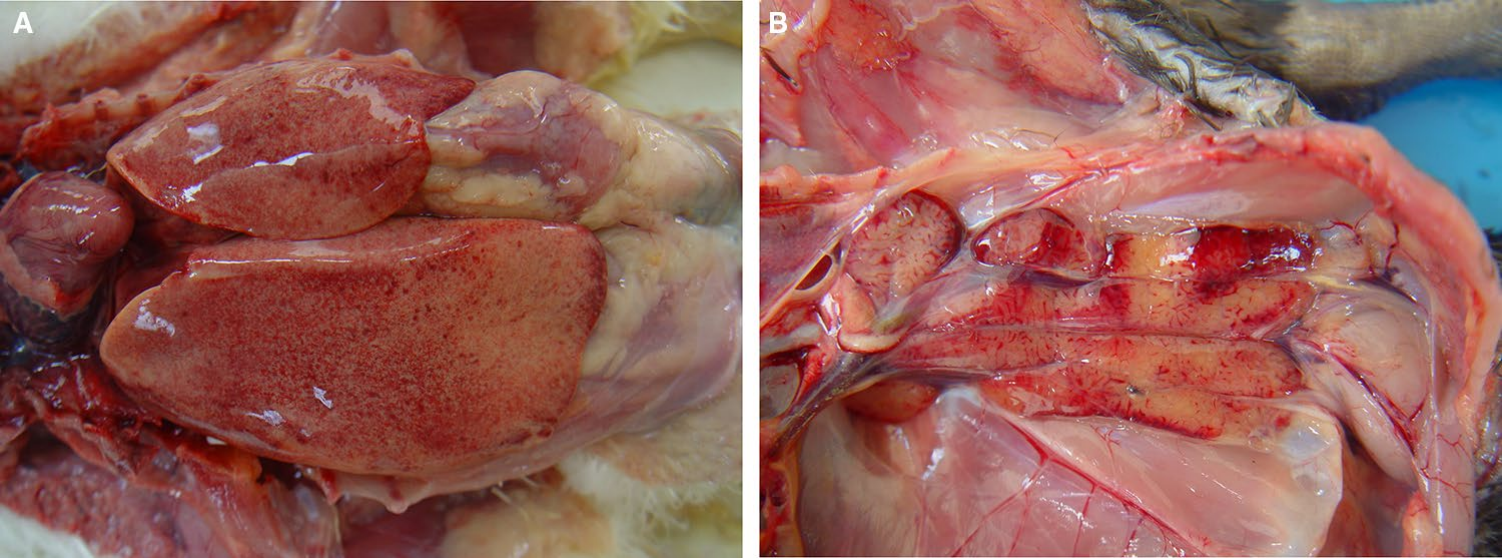
Gross lesion on the liver (A) and the kidney (B) of dead ducklings showing swelling and hemorrhage

Recently, several emerging outbreaks, characterized by swelling and hemorrhagic liver and kidney, have been threatening Muscovy duckling farms in Fujian, Zhejiang, Anhui and Guangdong provinces in China (Fig. 1). The morbidity has ranged from 40% to 55%, with a mortality rate of 35%-43%. To identify the causative agents, liver samples were collected from the affected Muscovy ducklings. Due to suspicion of possible bacterial infections, each sample was subjected to bacterial isolation using blood agar plates and tryptic soy agar plates as described by Liu *et al.* [8]. None of the samples were positive for bacterial isolation. The remaining samples were homogenized in Dulbecco’s modified Eagle’s medium (DMEM, Gibco, Grand Island, NY, USA). The tissue suspensions were subjected to three rounds of freeze-thawing before centrifugation at 5,000*g* for 15 min at 4 °C. The supernatants were then used for polymerase chain reaction (PCR) or reverse transcription PCR (RT-PCR) assays to detect avian influenza virus [9], avian paramyxovirus type 1, duck virus hepatitis A types 1 and 3 [10, 11], Muscovy duck reovirus [12], novel duck reovirus [13], avian Tembusu virus [14], duck astroviruses [15], Muscovy duck parvovirus [16], goose parvovirus [16], duck enteritis virus [17] and aviadenovirus (AdV) [18]. The samples were positive only for AdV.

Duck embryo fibroblasts (DEFs) were freshly prepared from 11-day-old Muscovy duck embryos and maintained in DMEM supplemented with 5% fetal calf serum (FCS, Gibco, Grand Island, NY, USA) for ~ 16 hours. AdV-positive samples were inoculated onto DEFs, and four virus isolates, designated as FJGT01, AHAQ13, ZJJH07 and GDMM10, were isolated successfully. Compared to normal DEFs, the infected cells became round and clustered like grapes (Fig. S1), which is a classical characteristic of the cytopathic effect in adenovirus-infected cells [19–22]. The titers of FJGT01, AHAQ13, ZJJH07 and GDMM10 were determined as 10^5.5^ TCID50/ml, 10^3.5^ TCID50/ml, 10^4.3^ TCID50/ml, 10^3.0^ TCID50/ml, respectively. For transmission electron microscopy (TEM) examination, the cell culture fluid of infected DEFs was harvested and then clarified by centrifugation at 8,000*g* for 30 min using a Ti19 rotor (Beckman Coulter, CA, USA) at 4 °C. The supernatant was collected, overlaid onto a 20% sucrose solution, and then centrifuged at 100,000*g* for 3 h at 4 °C in a Ti70 rotor (Beckman Coulter, CA, USA). The pellet was resuspended in TE buffer (10 mM Tris, pH 8.0, 1 mM EDTA, pH 8.0). The virus preparations were stained with phosphotungstic acid (Ted Pella Inc., Redding, CA, USA) and examined using TEM. Spherical particles ranging from 60 nm to 80 nm in diameter were observed (Fig. S2). These results indicated that the newly isolated viruses were AdVs.

The complete genomes of the identified were amplified using a set of specific primers (Table S1). PCR was carried out in a PCR system (C100 Touch^TM^ Thermal Cycler, Bio-Rad, Hercules, CA, USA). The PCR products were purified using a QIAquick Gel Extraction Kit (QIAGEN, Düsseldorf, Germany) and then cloned into pGEM®-T Easy Vector (Promega, Madison, WI, USA) according to the manufacturer’s instructions. After transformation of competent *Escherichia coli* strain DH5α (TaKaRa, Dalian, China) with these constructs, three positive clones for each PCR product were sequenced in both directions using an ABI 3730XL DNA Analyzer (Applied Biosystems, CA, USA) at BioSune Biotech Co., Shanghai, China. The genomic sequences of the AdV isolates were assembled using the Lasergene sequence analysis software package (DNASTAR Inc., WI, USA). The complete genomic sequences of FJGT01, AHAQ13, ZJJH07 and GDMM10 isolates were obtained and deposited in the GenBank database with the accession numbers MH777395, MH777396, MH777397 and MH777398, respectively. The full-length genomes of all four isolates were 43842 bp in length. The G+C content of 47.13% and the predicted ORFs were similar to those reported for other adenoviruses.

Sequence comparisons showed that the newly isolated AdVs shared nearly 100% genomic sequence identity and 100%, 99.8%-100%, and 100% amino acid identity in the hexon, fiber 1, and fiber 2 proteins with the first DAdV-3 isolate CH-GD-12-2014. However, all four isolates shared only 86.9% amino acid sequence identity and 76.6% nucleotide sequence identity in the hexon with DAdV-2 GR. The most significant difference between the new isolates and DAdV-2 GR was that the newly isolated AdVs contained fiber 1 and fiber 2 genes, whereas the DAdV-2 GR isolate only had one fiber gene. These data suggested that the newly isolated AdVs were DAdV-3 strains.

**Fig. 2:**
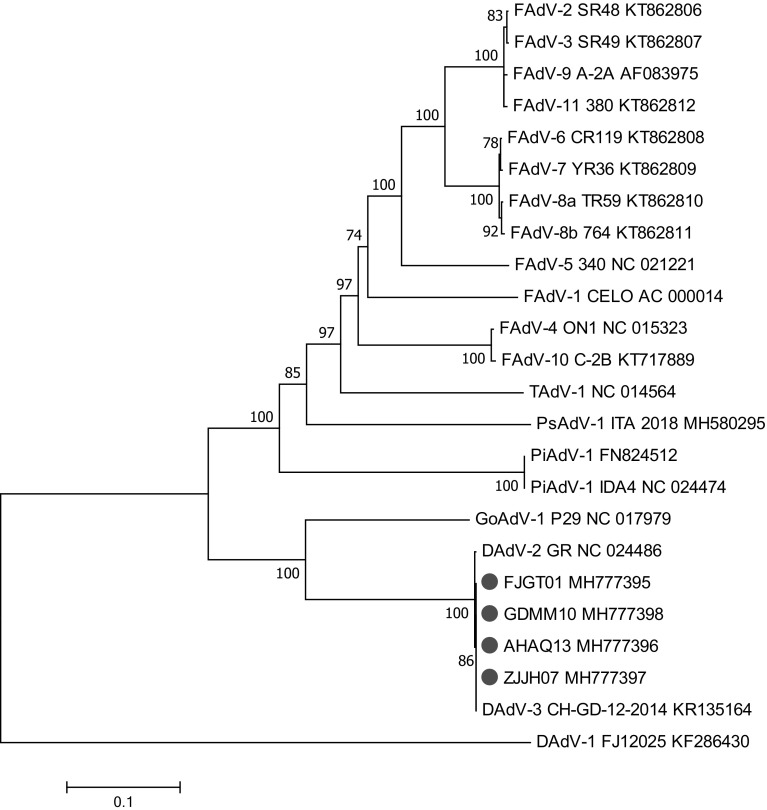
Phylogenetic analysis of duck adenoviruses and their counterparts based on the amino acid sequence of DNA polymerase. The tree was build using the neighbor-joining algorithm. Values at branches represent bootstrap support based on 1000 replicates. The new isolates are indicated by solid red circles

Phylogenetic distance based primarily on distance matrix analysis of the DNA polymerase amino acid sequence is an important standard for the classification of aviadenoviruses according to the Ninth Report of the ICTV [1]. A new adenovirus species can be established if the phylogenetic distance of the DNA polymerase amino acid sequence ranges from >5% to 15% in association with a lack of cross-neutralization if antisera are available [1, 23]. The phylogenetic distance based on the DNA polymerase amino acid sequence between the newly isolated DAdV-3 strains and DAdV-2 GR, the representative strain of the species *Duck aviadenovirus B*, was nearly zero (0.004), suggesting that the new isolates DAdV-3 FJGT01, GDMM10, AHAQ13, and ZJJH07 are members of the species *Duck aviadenovirus B* (Fig. 2).

To verify the agent, animal experiments were carried out. All animal studies were done in compliance with the regulations and guidelines of Fujian Academy of Agricultural Sciences for institutional animal care (No.1306129FAAS). Briefly, a total of 30 ten-day-old Muscovy ducklings were randomly divided into two groups (15 ducklings per group). The ducklings in the infection group were inoculated with 300 μl of FJGT01 containing 10^5.5^ TCID50/ml via the intramuscular route. Another group was inoculated intramuscularly with 300 μl of phosphate-buffered saline as a control. All of the Muscovy ducklings were monitored for 14 days. In the infection group, FJGT01 was highly virulent for Muscovy ducklings, with a mortality rate of 60%, which started on day 5 after inoculation. Gross lesions showed signs of swelling and hemorrhage in the liver and the kidneys of infected ducklings. All Muscovy ducklings in the control group remained clinically healthy.

**Fig. 3:**
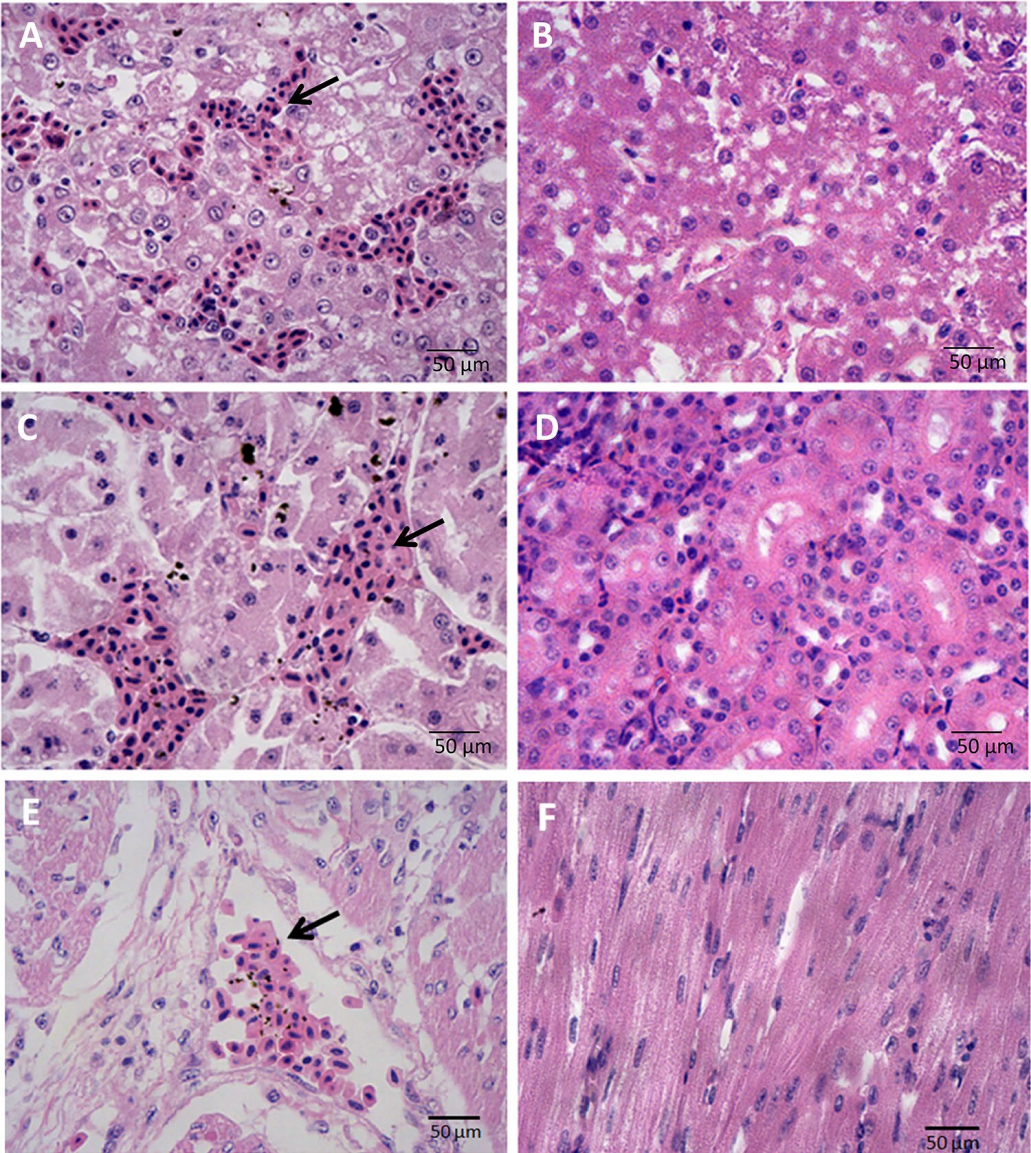
Histopathological analysis of tissues from dead Muscovy ducklings infected with DAdV-3 FJGT01. The liver (A, infected group), kidney (C, infected group), and heart (E, infected group), showed obvious hemorrhage (black arrows). B, D and E show the histopathological observations in liver, kidney and heart, respectively, of Muscovy ducklings in the control group (scale = 50 μm)

Tissues of the heart, liver, spleen, lung, kidney, brain, and pancreas of dead Muscovy ducklings were collected for histological examination. The tissues were fixed, cut into sections, and stained with hematoxylin and eosin (Fig. 3). Histopathological examination of the treated group revealed congestion in the liver, and the dilated sinusoid was full of red blood cells. In addition, hepatocytes were swollen and denatured with round or irregular vacuoles that appeared in the cytoplasm. The myocardial fiber was edematous, coarser, and slightly congested. Congestion was also observed in the kidney. The inflammatory cells were aggregated. Renal tubular epithelial cells were swollen and degenerated, and some epithelial cells were necrotic. However, there were no obvious histological lesions in the spleen, lung, brain, or pancreas. No significant histopathological damage was found in ducklings from the control group.

**Fig. 4:**
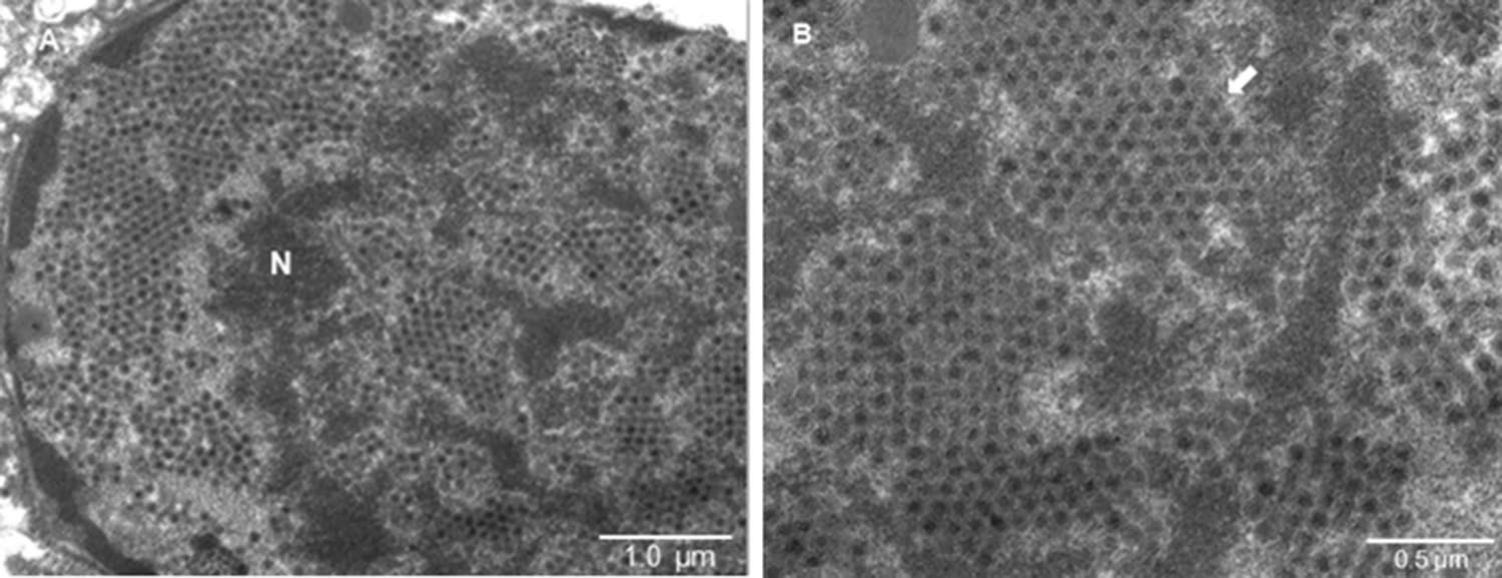
Electron microscopic view of the hepatic cell ultrastructure with accumulation of viral particles in the nucleus arranged in a crystal lattice (white arrow)

Liver samples were also collected for examination by TEM. The liver samples were embedded, and ultrathin sections were stained [24]. TEM demonstrated the presence of numerous icosahedral, nonenveloped viral particles measuring 60 nm to 80 nm in diameter. These virions were accumulated and arranged in crystal lattice formations in the nuclei of hepatocytes (Fig. 4), which is consistent with adenoviral infection [25–28]. This suggests that the liver is a tropic tissue of DAdV-3 and that viruses are synthesized and packaged in the nuclei of target cells. The structure, size, and lattice formation by these viral particles supported the diagnosis of an adenoviral infection.

In summary, in the present study, we isolated and identified DAdV-3 that was circulating in China. The newly isolated DAdV-3 strains could be classified as numbers of the species *Duck aviadenovirus B* in the genus *Aviadenovirus.*

## Electronic supplementary material

Below is the link to the electronic supplementary material.

Supplementary material 1 (TIFF 18138 kb)

Supplementary material 2 (TIFF 18548 kb)

Supplementary material 3 (TIFF 20500 kb)

Supplementary material 4 (TIFF 19954 kb)

Supplementary material 5 (TIFF 17893 kb)

Supplementary material 6 (DOCX 15 kb)
