# Supplementary figures and images for: Isolation and characterization of duck adenovirus 3 circulating in China
Source: Arch Virol. 2018 Dec 18;164(3):847–51. doi: 10.1007/s00705-018-4105-2 (PMC6394704; doi:10.1007/s00705-018-4105-2)

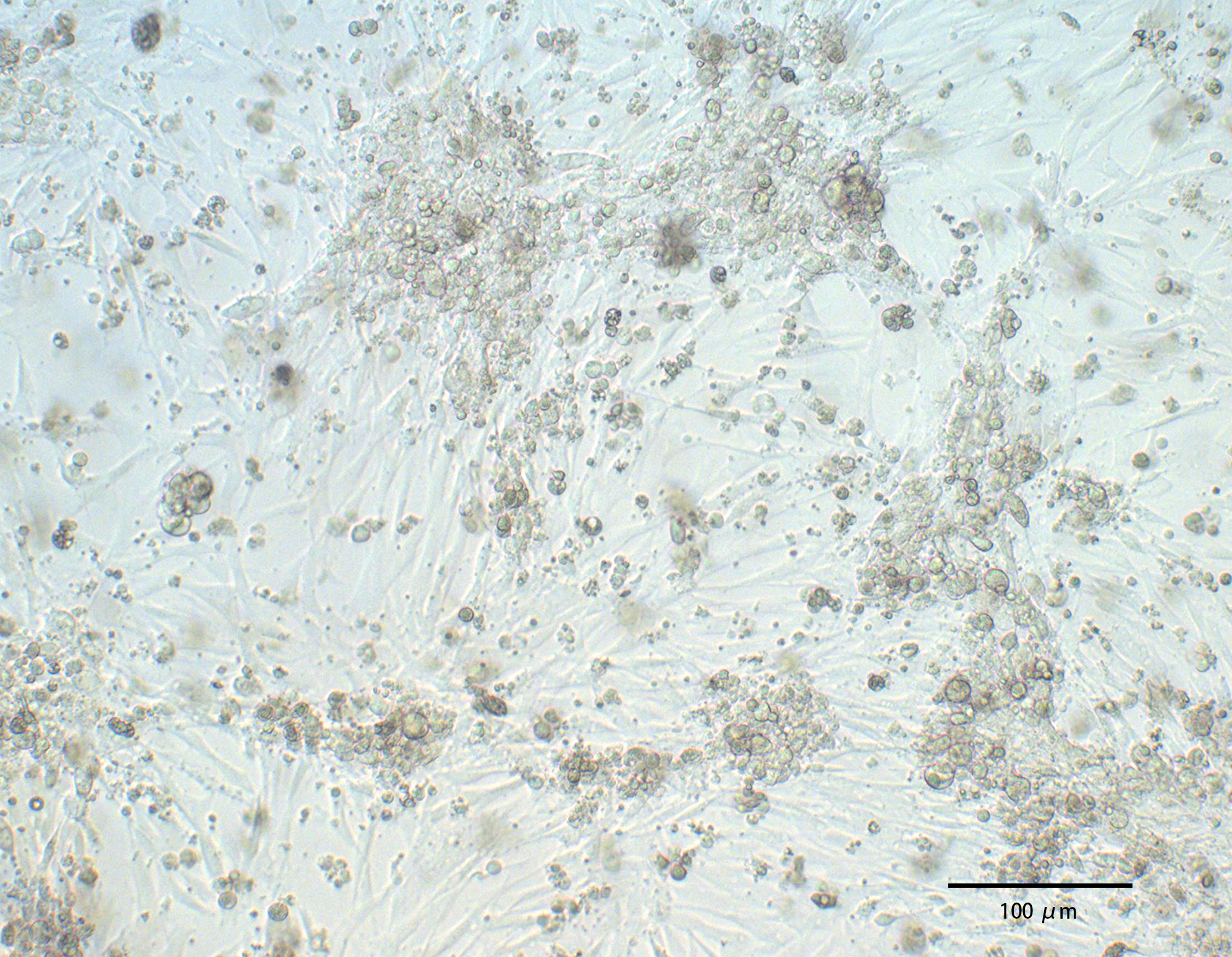

Supplement: Supplementary file 1 — Supplementary material 1 (TIFF 18138 kb) [file 705_2018_4105_MOESM1_ESM.tif]

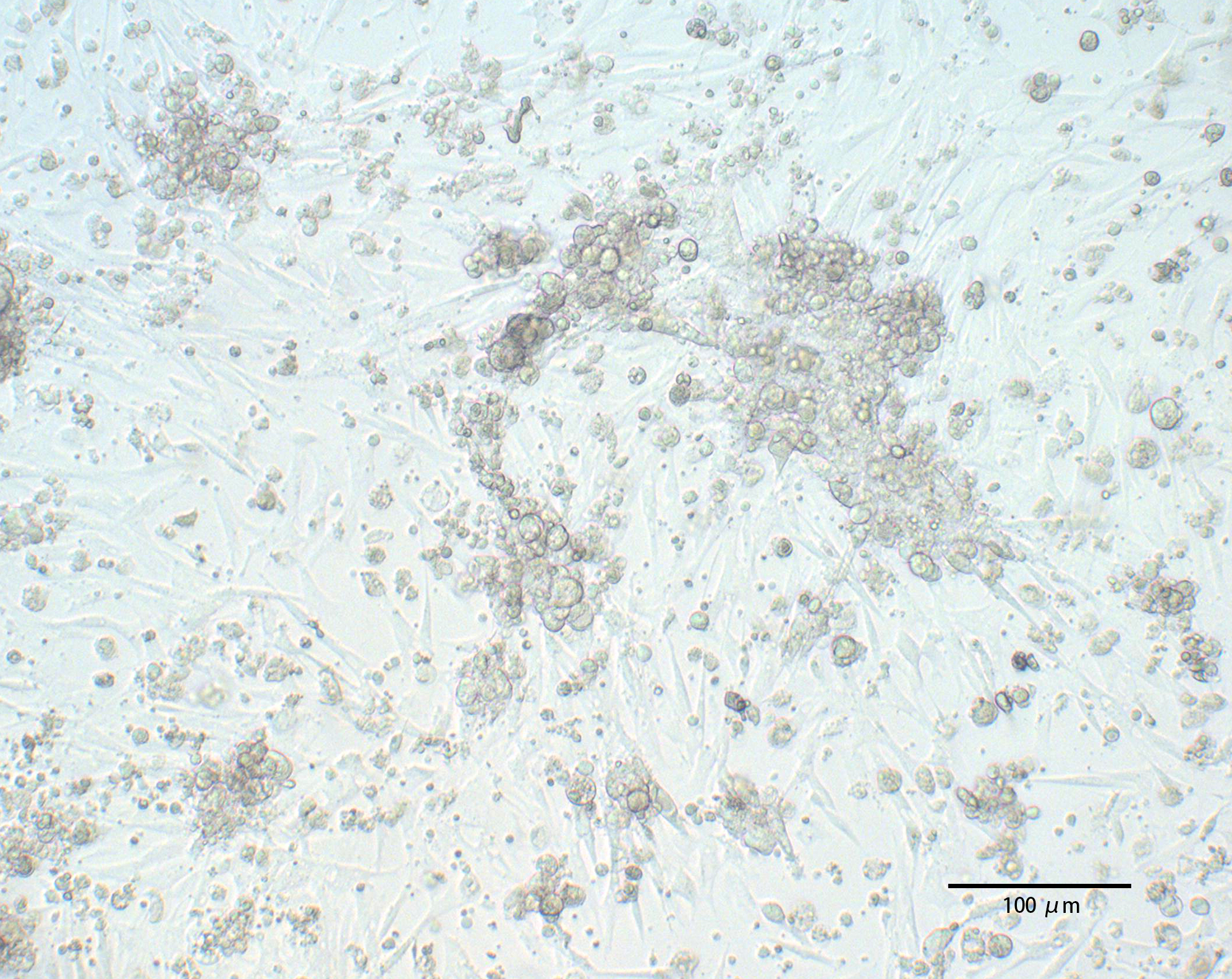

Supplement: Supplementary file 2 — Supplementary material 2 (TIFF 18548 kb) [file 705_2018_4105_MOESM2_ESM.tif]

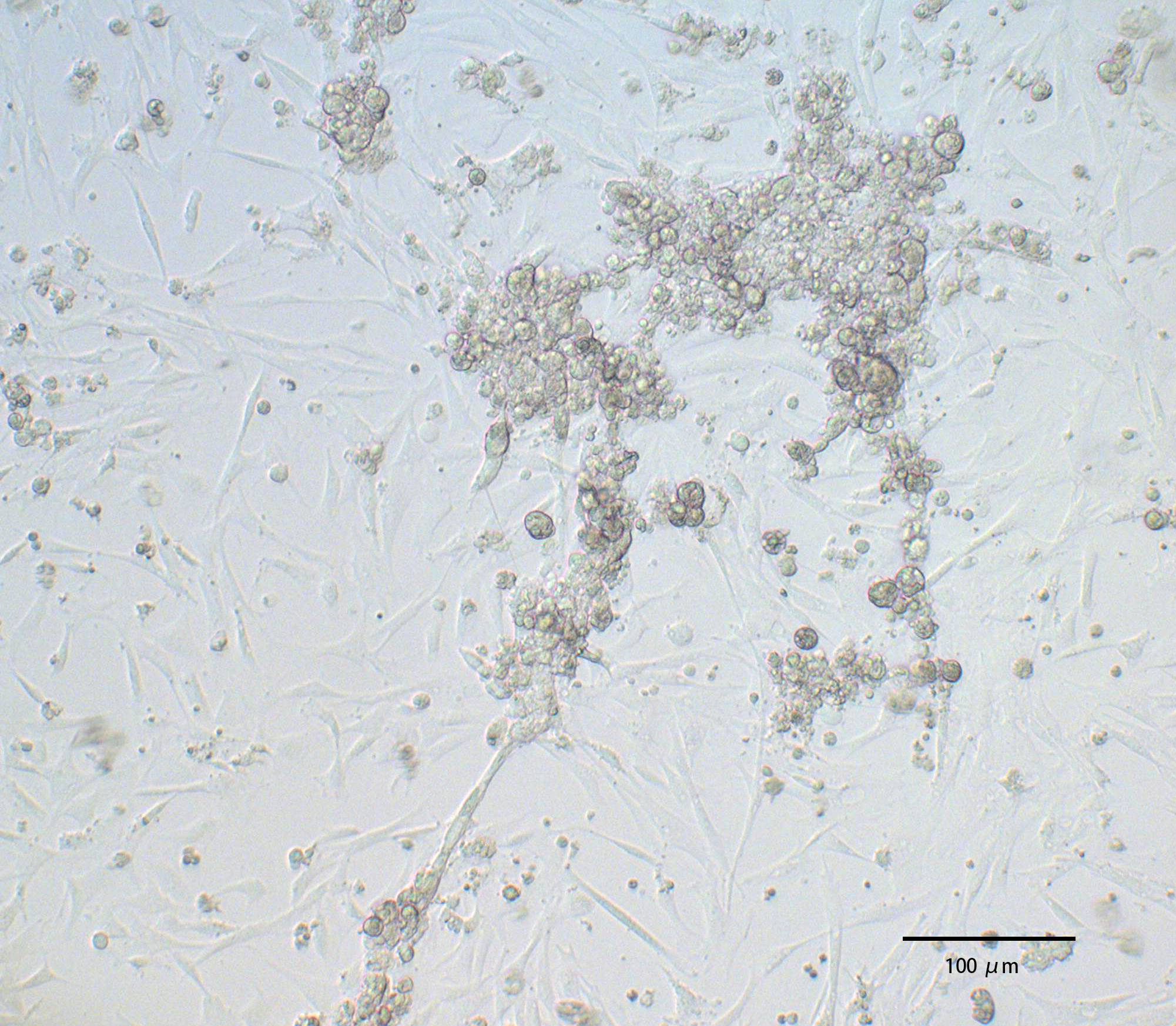

Supplement: Supplementary file 3 — Supplementary material 3 (TIFF 20500 kb) [file 705_2018_4105_MOESM3_ESM.tif]

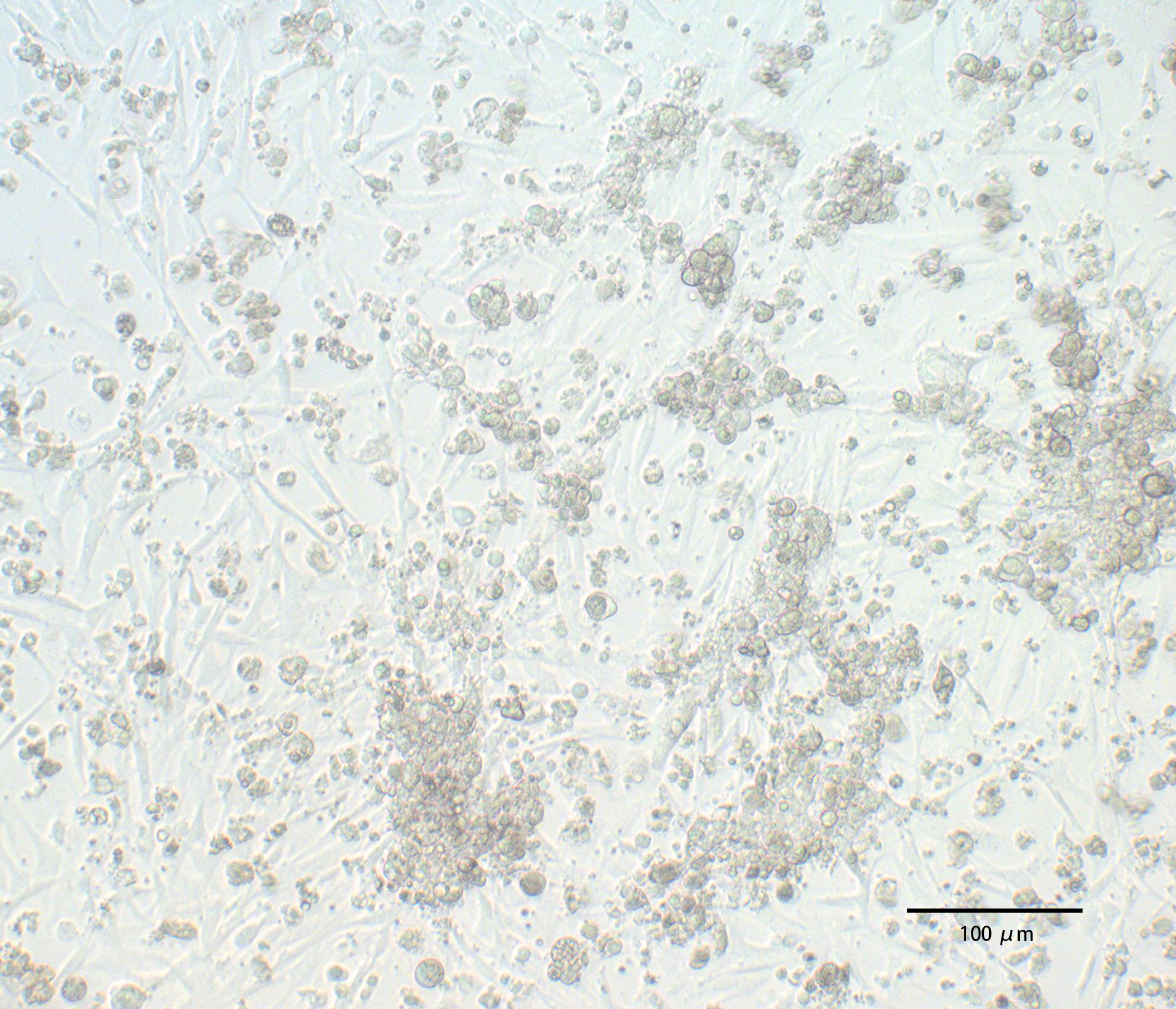

Supplement: Supplementary file 4 — Supplementary material 4 (TIFF 19954 kb) [file 705_2018_4105_MOESM4_ESM.tif]

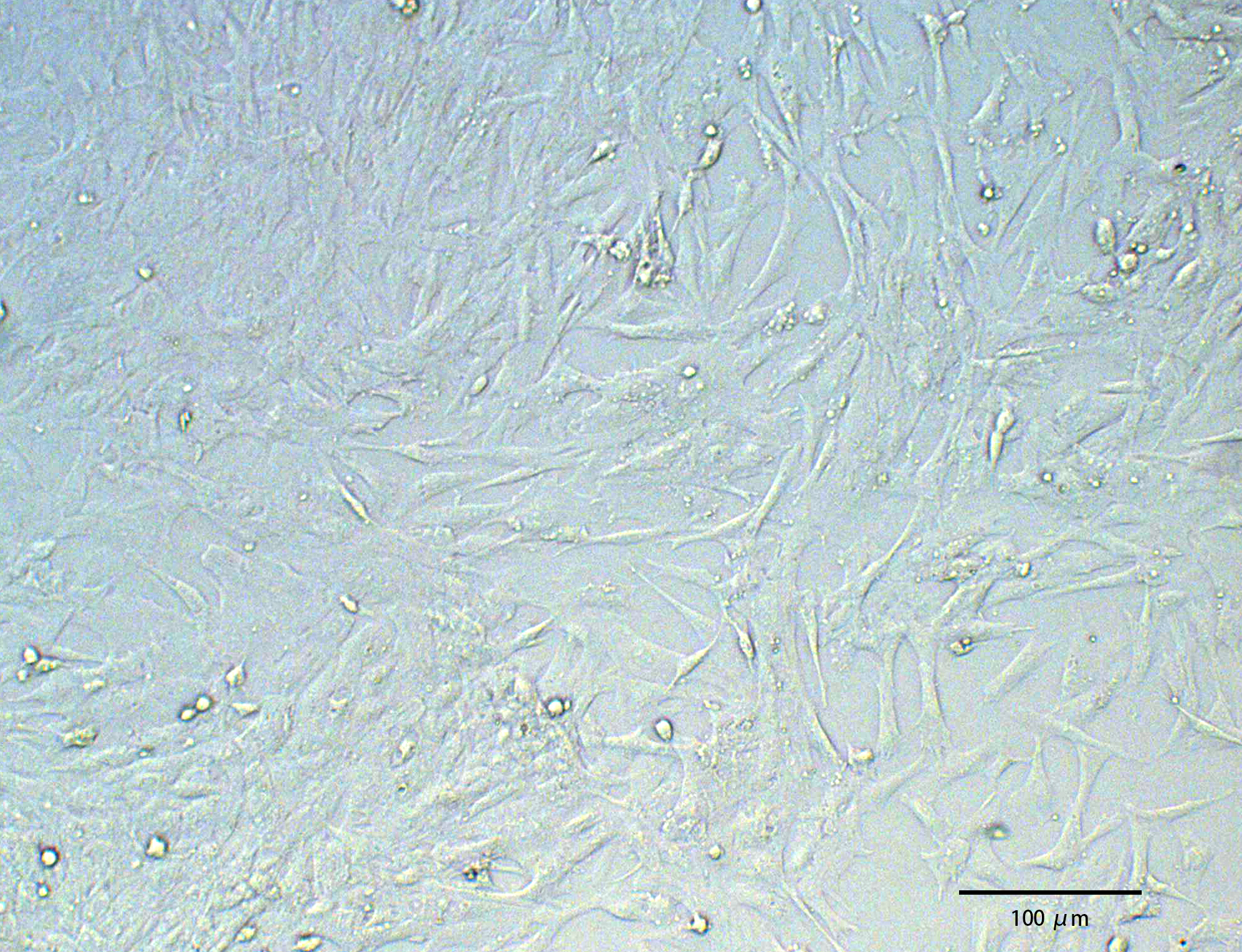

Supplement: Supplementary file 5 — Supplementary material 5 (TIFF 17893 kb) [file 705_2018_4105_MOESM5_ESM.tif]
