# Supplementary material for: Isolation and characterization of duck adenovirus 3 circulating in China
Source: Arch Virol. 2018 Dec 18;164(3):847–51. doi: 10.1007/s00705-018-4105-2 (PMC6394704; doi:10.1007/s00705-018-4105-2)
Supplement: Supplementary file 6 — Supplementary material 6 (DOCX 15 kb) [file 705_2018_4105_MOESM6_ESM.docx]

| **Table S1 List of primers for sequencing used in this study** | | |
| --- | --- | --- |
| Primer name | Sequence | Product size |
| AdV 1U24 | CATCATCATATATATACCATCGGT | 1739 bp |
| AdV 1718L22 | CCACATAAGTCCAAAAGTGGTC |  |
| AdV 1626U24 | ACTGGGTGGACAACCGATGAGTAA | 2020 bp |
| AdV 3622L24 | GGACTGTATCCATGCCTATCTTGC |  |
| AdV 3567U24 | CAGGTCGTCAAATTTTGTCACCAT | 2412 bp |
| AdV 5955L24 | TGGATCTCTGTCCAAACATTTACA |  |
| AdV 5874U24 | CTTCATTTTTGATGGCTTCAAACT | 1735 bp |
| AdV 7588L21 | TTATGCGGTCCATCTCCAAGC |  |
| AdV 7530U23 | ACTACCTTGGTGGTGTCCATGTT | 4247 bp |
| AdV 11754L23 | CAAGAACCGTCTACCGAGTCAGT |  |
| AdV 11685U23 | CTCTCGTCTGACGTTGTTGGACC | 3001 bp |
| AdV 14663L23 | AGTCGCGATCCTCGTCTAAGTCC |  |
| AdV 14456U21 | ACAGGCTCCATGAGCTCTCAC | 2295 bp |
| AdV 16730L21 | TTGAGCCAGAGTCCGTAAAGC |  |
| AdV 16657U21 | TCTTCCGAAAAGGAAACGCAC | 2902 bp |
| AdV 19535L24 | AGGTGTATGTTCCTGGCAGAAGTA |  |
| AdV 19466U24 | TAGGAAACAGCCGTTACTGTCAGT | 2874 bp |
| AdV 22316L24 | ACCTTTTCCACCTATGAGAGCTTC |  |
| AdV 21888U24 | TTCGGATACAGAGACTGGGTCCAC | 2944 bp |
| AdV 24808L24 | GCTCTTTTGCATCTCCTCGGTCAG |  |
| AdV 24688U24 | GAGGGCACGGTCAAAGTGTACTCG | 3063 bp |
| AdV 27730L21 | CCACGTTCAGCCGTCCGCTGT |  |
| AdV 27643U23 | GGCAGTCAGTTGGGGTTAAGCTG | 3203 bp |
| AdV 30825L21 | GCAGCAGGACCCCGAGGACAG |  |
| AdV 30755U23 | GATGGGGGCTCTAGGTGGGATCG | 3428 bp |
| AdV 34158L25 | TGCAGGAATTGGGTGTTCAAAAAGA |  |
| AdV 34028U25 | TTCACAGGCTGTAATGTTCTGCTCC | 3103 bp |
| AdV 37106L25 | TTGGAGTCAATGGACATGGCTACTA |  |
| AdV 37029U25 | ATATGGAGCCTTAGTGTAACAATGC | 3114 bp |
| AdV 40118L25 | GAATTCTGCCTTGATTGTCTTTCTG |  |
| AdV 40016U25 | CTTAGGACTCCAAACCCAAATAGAT | 2763 bp |
| AdV 42760L19 | TGCCATGCGCTACGCTAAG |  |
| AdV 42716U19 | CATTCGTCATGCTTGGTGC | 1085 bp |
| AdV 43778L23 | ATTTTTACTAGGGGGTCAAAGGT |  |
| Primers were named based on nucleotide position located in the CH-GD-12-2014 (GenBank: KR135164). | | |
